# An optimal set of features for predicting type IV secretion system effector proteins for a subset of species based on a multi-level feature selection approach

**DOI:** 10.1371/journal.pone.0197041

**Published:** 2018-05-09

**Authors:** Zhila Esna Ashari, Nairanjana Dasgupta, Kelly A. Brayton, Shira L. Broschat

**Affiliations:** 1 School of Electrical Engineering and Computer Science, Washington State University, Pullman, Washington, United States of America; 2 Department of Mathematics and Statistics, Washington State University, Pullman, Washington, United States of America; 3 Department of Veterinary Microbiology and Pathology, Washington State University, Pullman, Washington, United States of America; 4 Paul G. Allen School for Global Animal Health, Washington State University, Pullman, Washington, United States of America; Centre National de la Recherche Scientifique, Aix-Marseille Université, FRANCE

## Abstract

Type IV secretion systems (T4SS) are multi-protein complexes in a number of bacterial pathogens that can translocate proteins and DNA to the host. Most T4SSs function in conjugation and translocate DNA; however, approximately 13% function to secrete proteins, delivering effector proteins into the cytosol of eukaryotic host cells. Upon entry, these effectors manipulate the host cell’s machinery for their own benefit, which can result in serious illness or death of the host. For this reason recognition of T4SS effectors has become an important subject. Much previous work has focused on verifying effectors experimentally, a costly endeavor in terms of money, time, and effort. Having good predictions for effectors will help to focus experimental validations and decrease testing costs. In recent years, several scoring and machine learning-based methods have been suggested for the purpose of predicting T4SS effector proteins. These methods have used different sets of features for prediction, and their predictions have been inconsistent. In this paper, an optimal set of features is presented for predicting T4SS effector proteins using a statistical approach. A thorough literature search was performed to find features that have been proposed. Feature values were calculated for datasets of known effectors and non-effectors for T4SS-containing pathogens for four genera with a sufficient number of known effectors, *Legionella pneumophila*, *Coxiella burnetii*, *Brucella* spp, and *Bartonella* spp. The features were ranked, and less important features were filtered out. Correlations between remaining features were removed, and dimensional reduction was accomplished using principal component analysis and factor analysis. Finally, the optimal features for each pathogen were chosen by building logistic regression models and evaluating each model. The results based on evaluation of our logistic regression models confirm the effectiveness of our four optimal sets of features, and based on these an optimal set of features is proposed for all T4SS effector proteins.

## Introduction

The type IV secretion sytem (T4SS) is a complex made up of proteins which deliver DNA and proteins to the host cell. Detection of the T4SS in a genome is relatively straightforward, as most of its genes can be detected through sequence identity using BLAST searches or predictive software [1, 2]. On the other hand, the proteins it secretes pose a much greater challenge. Proteins secreted by the T4SS are known as effectors and are agents of virulence and pathogenesis. They change the environment of the cell to be more hospitable for the bacterial pathogens allowing replication of the bacteria [3]. The importance of effector proteins is understood, but for the majority of effectors the more significant question of how they actually function remains a mystery. However, before function can be studied, effectors must be identified, and this is still a major challenge as experimental identification and verification is costly both in terms of time and money. In addition, effectors tend to be species specific, and it is much more difficult to detect them than the structural components of the T4SS for each species. With the advent of machine learning methods, researchers have turned to scoring methods [4] and machine learning algorithms [5–8] to predict effector proteins from the genomes or proteomes of pathogens. If prediction is known to be highly accurate, the process of experimental verification can be performed much more efficiently.

Several T4SS effector prediction algorithms have been published recently. Burstein et al. [7] used a machine-learning approach to consider *Legionella pneumophila* and predicted and validated 40 new effector proteins while Wang et al. [8] focused on *Helicobacter pylori*. The method by Meyer et al. [4] was first used with the effector dataset of *L. pneumophila*, strain Philadelphia, and then used for several other proteobacterial pathogens. The algorithms in these studies used sets of features, which are measurable characteristics and properties of protein sequences. Each algorithm employed a different set of features for effector prediction, and the different sets had either some or no features in common. This raised the issue of which feature set should be used to develop a new machine learning model. Also, while both [4] and [5] claim to predict effector proteins in T4SS pathogens, when we used their programs for the rickettsial pathogen *Anaplasma phagocytophilum*, which has a T4SS and only three validated effector proteins, the former predicted 20 effector proteins and the latter predicted 81. However, only one protein was common to both of them which is a known effector protein [9]. We conclude that the probable reason for the large discrepancy in the results is the difference in the feature sets used in the algorithms which are orthogonal to each other—that is, none of the features used by the two algorithms are shared. It should be noted that all features used in these works are listed in S1 Table, and the two different feature sets are identified. As a result of our analysis, we were motivated to study the effectiveness of all different features proposed in previous studies and to select the best features for effector prediction. This is the first study of its kind, i.e., it is the only analysis performed to determine the best features for predicting T4SS effectors.

Effector proteins are different among different pathogens [1] and, as such, the signals for transconductance via the T4SS apparatus are likely to differ. In this paper, we present a statistical study of the protein characteristics or features used to recognize effectors for several T4SS pathogens with the goal of identifying an optimal set that will potentially work well for all T4SS pathogens of interest. We performed a literature search for all features that had been previously used in scoring or machine learning approaches to predict T4SS effectors and compiled an extensive list of these features. The gathered features are related to the different characteristics of protein sequences including: chemical properties such as hydropathy and charge; structure and composition of sequences such as the presence of different domains, amino acid composition, and the position-specific scoring matrix (PSSM) profile of protein sequences; and the topology of the sequences such as their secondary structure. The complete list of features can be found in S1 Table. Also, we have provided the references from which we extracted each feature. We gathered a total of 51 features, but because four of them were vector features (for example, amino acid composition is a vector feature with 20 features because there are 20 different amino acids) and we used each of their elements as a separate feature in our analysis, we ended up with 1027 features.

Because pathogens in the Alphaproteobacteria and Gammaproteobacteria classes with T4SS effectors have relatively high rates of T4SS and are the best studied [1], for our dataset, we searched the literature for organisms in these two classes for confirmed effectors. We chose to consider *L. pneumophila*, *Coxiella burnetii*, *Brucella* spp, and *Bartonella* spp based on their number of effectors, 317, 86, 16, and 9, respectively. After generating datasets of confirmed effectors and non-effectors for the four pathogens, we calculated the value of each feature for all four datasets. By value of each feature, we mean the number associated with each feature after presenting it as a measurable property. Details on the features and how they were calculated are presented in [10]. Feature values vary in range and can be binary or continuous. For instance, presence of a region or domain is indicated by a binary value of 0 or 1, where 1 shows it is present, and chemical properties such as hydropathy are given by the calculated value of the protein sequence, which is continous. Also, we represent the secondary structure of proteins by the percentage of the particular structure present in the sequence. After feature values had been calculated, we began our statistical study by ranking and filtering features based on their p-values using a t-test. Next we normalized feature values and used principal component analysis (PCA) and then factor analysis for dimensional reduction and elimination of any correlation between features. Finally, using a fast backward feature selection method, we built logistic regression models to select an informative set of features that works well as a group of predictors for prediction of T4SS effectors for each genus. Based on the results of the four different feature sets, we were able to establish an optimal feature set for determining T4SS effector proteins of interest to researchers.

## Materials and methods

**Fig 1:**
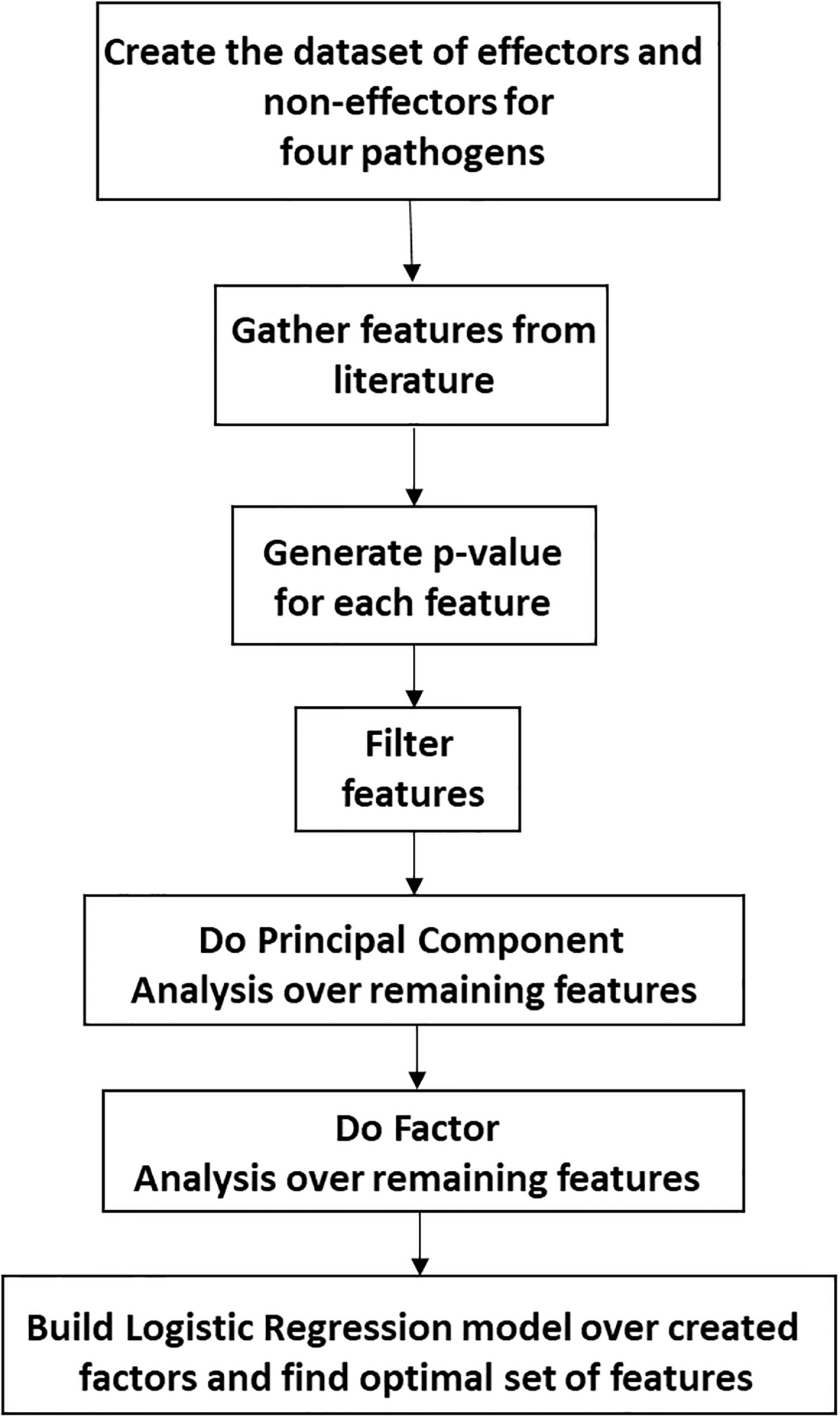
Workflow used to identify optimal features for predicting T4SS effector proteins.

A workflow of the methods used in this paper is shown in Fig 1. Each step in the workflow is described below; details of some of the steps are given in an earlier paper [10].

### Effector and non-effector datasets

Our goal was to determine an optimal set of features for prediction of all T4SS effector proteins, and as such, we decided to work with various pathogen datasets. However, to enable this, it was necessary to have a sufficient number of confirmed effector proteins. For our first step we searched through previous studies that had been done to verify T4SS effectors. Because pathogens in the Alphaproteobacteria and Gammaproteobacteria classes with T4SS, are of interest to many researchers, we searched the literature for organisms in these two classes for confirmed effectors. For Gammaproteobacteria, we found 317 effectors for *L. pneumophila* [11–31] and 86 effectors for *C. burnetii* [32–36]. For Alphaproteobacteria we found a total of 16 effectors for *Brucella abortus* and *Brucella melitensis* [37, 38] and a total of 9 effectors for *Bartonella henselae* and *Bartonella tribocorum* [39]. Next we used the non-effector dataset created by Zou et al. to build our own datasets [5]. In [5], protein sequences from the whole genome of 10 pathogens with T4SS, which have homologous genes to *E. coli*, were gathered, and the sequences that were highly similar to those in *E. coli* were selected using BLAST. Next the ones that were orthologous or paralogous were eliminated to reduce redundancy, leaving them with their non-effector dataset. More details concerning their method can be found in [5]. We downloaded the non-effector dataset from their website (http://bioinfo.tmmu.edu.cn/T4EffPred) and used the sequences related to our four genera of interest using 554, 95, 32, and 17 non-effector sequences for *L. pneumophila*, *C. burnetii*, *Brucella* spp, and *Bartonella* spp, respectively. The effectors and non-effectors used in this study are listed in the supporting information in S1 through S8 Files.

### Features and feature evaluation

The second step in this work involved reviewing the literature and gathering the features for predicting type IV effectors proposed previously. As such, we reviewed the literature that focused on predicting T4SS effectors using scoring or machine learning methods [4–8, 40–42] and selected all features relevant to the protein sequences for effectors and non-effectors in this study. The complete list of features used is given in S1 Table as supporting information. An explanation for each feature as well as the reference in which each feature was introduced is included in this table. The features are related to different chemical properties (different hydropathy measures as well as polarity, charge, basicity, molecular mass, and iso-electric point measures); structure (presence of various regions and domains such as coiled coil domain, Ank domain, as well as PSSM profile of protein sequences); composition (amino acid composition and dipeptide composition of amino acids); and topology of the protein sequences (percentage of secondary structure types). Descriptions of each feature along with software, programs, and tools used to calculate their values [43–48] can be found in [10].

### Feature selection filtering using *t*-test

In this step we used a filtering feature selection approach to eliminate less informative features. For this purpose, the *t*-test was used over the dataset of known effectors and non-effectors for each feature, and the calculated p-values associated with each feature were stored. The p-values represent the significance of each feature with lower p-values indicating a higher potential for use in our machine learning classifier. Finally, we eliminated less important features by filtering out those with higher p-values than the chosen threshold. To choose a threshold for p-values, we used Bonferroni correction resulting in a cut-off value of 0.0009. It should be noted that the most significant features had p-values on the order of 10^−100^ and the least significant ones had p-values of approximatley 0.9. Details for this step are described in [10], and the results are discussed in the Results and Discussions section of this paper.

### Principal component analysis

To this point and in our earlier work [10], we have chosen features for each type of bacteria by filtering out less important features based on the *t*-test. However, more sophisticated statistical methods can be used to determine how selected features might work together to predict T4SS effectors and which group is most effective. In addition, correlation between different features can be eliminated to avoid redundancy using a dimensional reduction method.

Toward this end, we used principal component analysis (PCA) for dimensional reduction of the number of features. PCA finds the features that have the largest amount of variance from other features. First we normalized continuous feature values to be on the same scale. Then we performed PCA using *Minitab* 17.1.0 software (http://www.minitab.com). In PCA the eigenvectors, or principal components, for the correlation matrix of the features are calculated. Features are projected in the directions of the calculated eigenvectors and are called factors. Since, eigenvectors are orthogonal to each other, factors are orthogonal as well and, thus, have no correlation. This eliminates redundancy. Next by calculating the eigenvalue associated with each factor, we can find the variance between the factors and we consider those that have the largest variance. For this purpose, a scree plot is used. A scree plot displays eigenvalues as functions of factors, or principal components, in descending order, i.e., the largest eigenvalues represent the greatest variance. For our work, we considered factors that had eigenvalues greater than 1, which is the value commonly used, and ignored all others. In this way we obtained the number of effective factors for each pathogen.

### Factor analysis

In conjunction with the PCA performed in the previous step, factor analysis was used. The idea behind the use of factor analysis was to remove features by finding similar underlying patterns of features that represent a so-called latent feature that cannot be measured directly. For example, a socioeconomic status latent feature might be represented by the features net worth, occupation, and number of vacation homes. Mathematically, each feature value is given as a sum of factor loadings times factors with the number of feature values greater than the number of factors, and factor loadings can be thought of as how much features correlate with factors. To obtain the factor loadings, *Minitab* was used with the number of factors determined previously by means of PCA. As mentioned in the previous section, we performed PCA and factor analysis over continuous features. Thus, we retained the factors determined from factor analysis and combined these with our binary features as separate factors to form our final factor set for use in our logistic regression model. Also, we retained the factor loadings to determine which features to retain at the end of our study based on the selected set of factors. For example, if socioeconomic status is represented by one factor, and the factor loadings for net worth, occupation, and number of vacation homes are 0.64, 0.60, and 0.70, we might remove net worth and occupation from our set of features because number of vacation homes is sufficient to represent socioeconomic status.

### Logistic regression feature selection

After reducing the dimensions of our predictor set and calculating the effective factors, we used them to build a binary logistic regression model for using a fast backward feature selection method. As we have two classes of responses (effector and non-effector), binary logistic regression is a suitable analysis method. Logistic function input can be any real number and its output takes a value between 0 and 1, representing the probability of being an effector. The logistic function format is given by Eq (1).

$$\begin{array}{c} f\left(x_{1},x_{2},\ldots \right)=\frac{e^{\left(a_{1}*x_{1}+a_{2}*x_{2}+\ldots +b\right)}}{e^{\left(a_{1}*x_{1}+a_{2}*x_{2}+\ldots +b\right)}+1} \end{array}$$ (1)

For this step, we used *Minitab* software to build a logistic regression predictor model for testing our calculated factors and to determine which ones were the most effective based on the built model. We used factors as independent variables and constructed a logistic regression model for each of the four bacteria types. Also, the Hosmer-Lemeshow test, which is a goodness-of-fit test, was used to evaluate our model to ascertain how well our predicted model matches the expected model and predicts the effectors. It works by grouping the input dataset of effectors and non-effectors based on estimated probabilities of being an effector. Most software groups data into deciles, using 10 percent of the data in each group, which is the case for our work. Then the model is used to predict whether they are effectors or non-effectors. The percentage of expected and observed results that are in concordance are then calculated.

Considering the logistic function in Eq (1), we see that it associates a coefficient with each independent variable, and the ones with larger coefficients are more effective in the model. First, we built our logistic regression models such that we did not have complete separation between effectors and non-effectors based on the factors which happens readily for small datasets. In this way we were able to discern the most informative factors and eliminate the least informative ones. We then built a logistic regression model again and evaluated the effectiveness of the remaining factors. We continued until the concordance rate from the Hosmer-Lemeshow test stays acceptable and greater than 90%. In this way, the set of factors working most effectively to predict effector proteins was selected.

In the final step, as discussed in the factor analysis section, we used the factor loadings to determine the set of original features that were selected from the selected factors. If we assume that each original feature is represented by the factor with the greatest loading, we know the set of original features that each factor represents. In this way, we created the group of selected features for each of the four types of pathogens.

## Results and discussion

To understand what features are important for T4SS prediction, we first used a feature selection filtering method over our feature set for four pathogens similar to the method applied in [10] and created a ranked set of the remaining features based on their importance. Afterwards we used PCA and factor analysis over the features to reduce their dimensions and also to eliminate any correlation and redundancy among them. These steps led to generation of factors that were used in building logistic regression models for the purpose of selecting an informative group of features.

**Fig 2:**
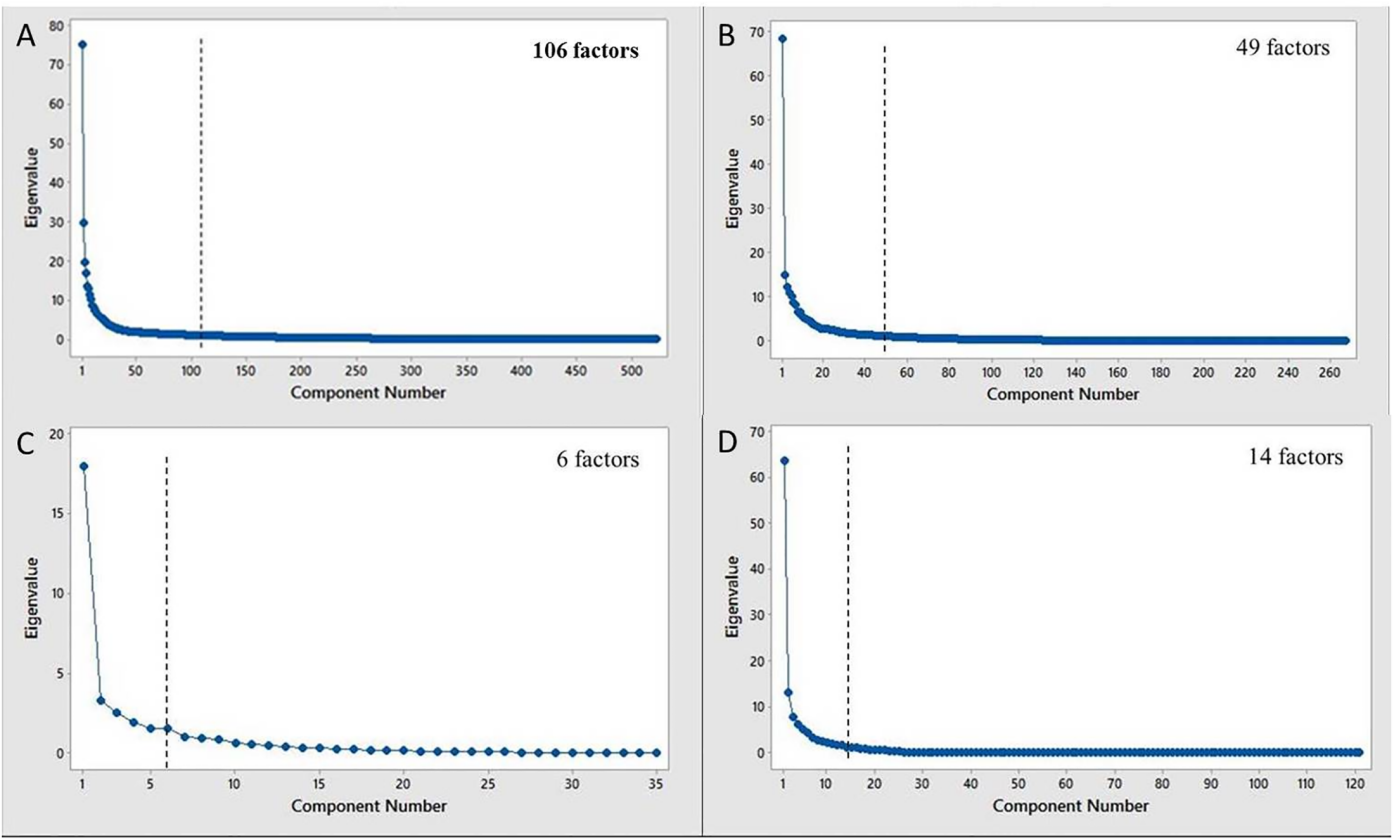
The PCA scree plots show the values of an eigenvalue versus its factor or principal component number. The dashed vertical line in each plot shows a cut-off value of one for the eigenvalue. Factors to the right of each line were discarded. The number of factors used for each pathogen is given in the top right corner of each plot. (A) *L. pneumophila*, (B) *C. burnetii*, (C) *Brucella spp*, and (D) *Bartonella spp*.

To determine the number of effective factors to be considered, PCA analysis was used and scree plots for each pathogen were created. A scree plot displays the eigenvalues associated with factors in decreasing order plotted versus factor number. We can see the scree plots for our four bacteria types in Fig 2. As described before, to determine the number of necessary factors, we considered the number of eigenvalues which were greater than 1 using the scree plots. As shown in Fig 2, the selected number of factors are 106, 49, 6, and 14 for *L. pneumophila*, *C. burnetii*, *Brucella spp*, and *Bartonella spp*, respectively. Using a cut-off of 1 for the eigenvalues is a conservative approach for selecting the number of factors because it allows us to retain most of the variability of the data without suffering from the redundancy caused by extreme pairwise correlations. Thus, dimensions are drastically reduced while the selected factors, explain 84%, 89%, 84%, and 95% of the total variability in the feature sets for the named pathogens, respectively. As such, we keep most of the variability in our datasets, even after selecting a subset of factors among all the factors, which shows that it will not cause significant loss in fit. While there is a loss in information using this approach, PCA allows us to reduce dimensionality, and the use of factor analysis makes our newly created factors interpretable. Using this approach was conservative in the sense that if a feature was very significant, it was included in our factors.

Next, factor analysis was used and a set of final factors were generated. The next step was building a logistic regression model for each pathogen. We calculated the coefficients of each factor in the logistic function as well as the p-values associated with the null hypothesis stating that by setting the coefficient of a factor to zero, the model will not change significantly and so there is not a significant association between a factor and the expected outputs. Thus, by removing the factors with greater p-values and keeping the ones with p-values that were approximately zero, we eliminated the less informative factors and rebuilt the model with the remaining factors.

As mentioned, the Hosmer-Lemeshow goodness-of-fit test was performed over the four final logistic regression models to verify their effectiveness. This test divides the input dataset of effectors and non-effectors into 10 groups according to their predicted probabilities of being an effector and predicts whether they are effectors or non-effectors using our model. Finally, it calculates the percentage of expected and observed results that are in concordance. The achieved results of concordant percentages are presented in Table 1 for the four pathogens. The results are significant and show how well our logistic regression models work.

**Table 1:** Hosmer-Lemeshow goodness-of-fit test: Concordant percentages between effector predictions using our built logistic regression models and known effectors.

Concordant percentage			
*L. pneumophila*	*C. burnetii*	*Brucella spp*	*Bartonella spp*
97.8	95.8	98.4	98.0

In order to evaluate our final logistic regression models further, we decided to consider residuals, which indicate the difference between the true and predicted values using the model (by value, we mean the probability of being an effector). Using *Minitab*, deviance residuals were considered for each data point where the residual is equal to -2 times the logarithm of the absolute difference between the predicted probability and 1 (if it is not an effector) or 0 (if it is an effector). For a good model, the residual of a data point should be close to zero.

**Fig 3:**
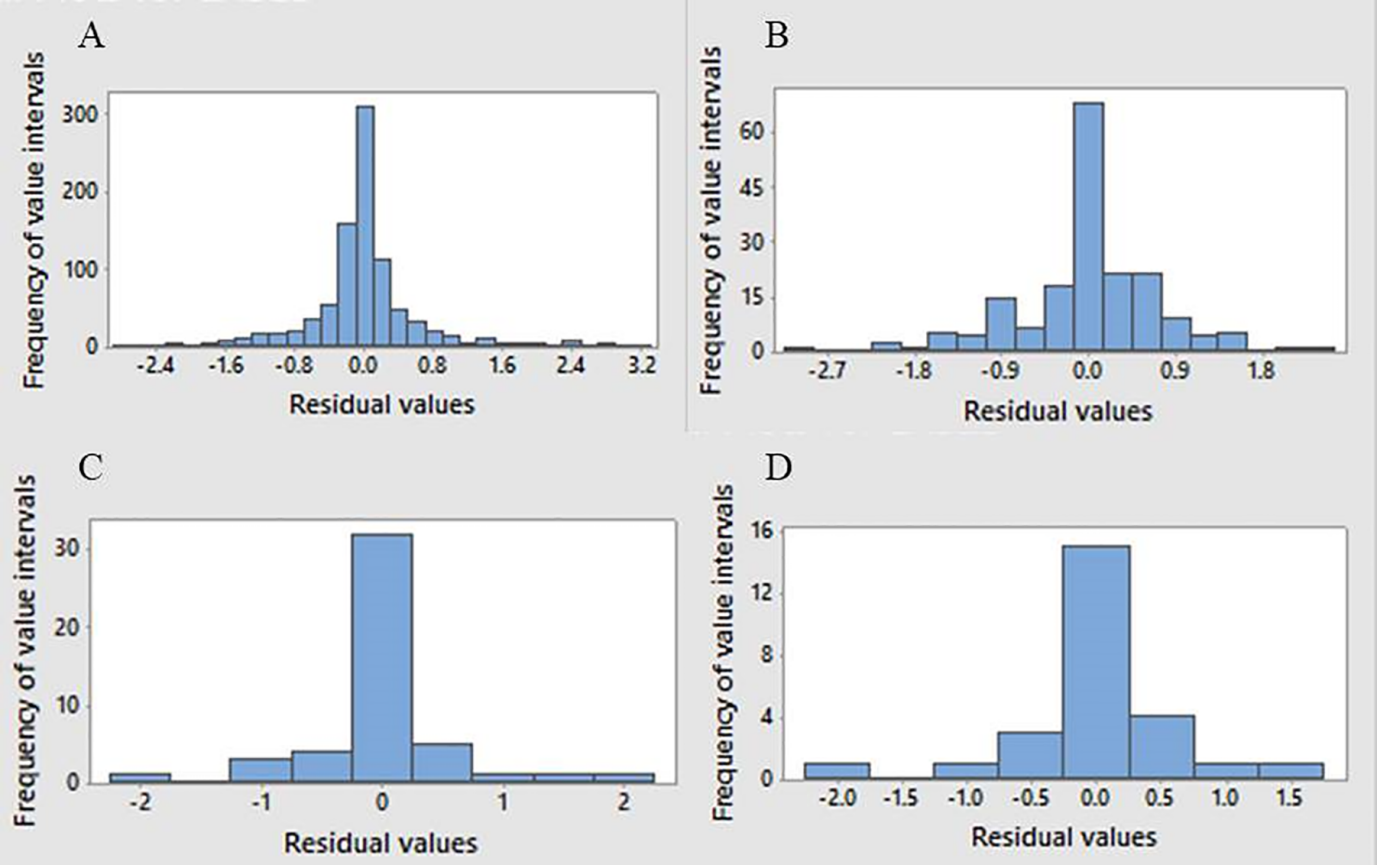
Histogram of residual values showing the frequency of each value interval versus residual values. Residuals represent the difference between true and predicted values using our final logistic regression models. It can be seen that residuals are concentrated around zero and have a normal distribution and also are not skewed and contain no outliers. (A) *L. pneumophila*, (B) *C. burnetii*, (C) *Brucella spp*, and (D) *Bartonella spp*.

Residual histograms are plotted for our four logistic regression models and shown in Fig 3. An examination of this figure shows that for all four pathogens the residuals are concentrated around zero and have normal distributions and, thus, there are not many outliers.

Based on the analysis done on the final logistic regression models, we can conclude that for four pathogens, the group of selected features work effectively together.

The next step was to revert from factors to our original features using the saved factor loadings. Using the absolute value of the largest factor loading for each feature enabled identification of the factor associated with the feature which, in turn, showed which factor represents which group of features. As a result, we converted the group of final factors to the group of final selected features by substituting each factor with the features it represents. This was repeated for all four pathogens and the sets of selected features for each one are shown in S2 to S5 Tables. In these tables, elements of vector features are considered as separate features and we can see which elements are selected as effective.

Finally, for T4SS effector protein prediction, we created a set of the union of all selected features presented in S2 to S5 Tables and made a list of selected effective features for prediction of T4SS effectors. The list is presented in S6 Table as supplementary information.

Based on the calculated p-values after using *t*-test, we created a ranked set of features based on their effectiveness for our four types of bacteria, which are shown in Table 2. The numbers in each column show the rank for each feature for each bacterium. The top part of Table 2 shows the four vector features which are ranked based on the percentage of elements that were selected after applying our filtering method. We conclude that amino acid and PSSM composition are the two most predictive vector features while dipeptide composition is the least. The middle part of Table 2 represents the ranked set of other features, while the features in the lower part of the table are ranked but were not selected for any of our bacterial pathogens.

**Table 2:** Features in different steps: Features are ranked based on p-values and the ones selected using filtering method are underlined for each pathogen. The ones selected using logistic regression are in bold. The last column shows the selected features for T4SS prediction. Upper part of table shows vector features and the bottom part shows the features that have not been selected for any of the pathogens.

No.	Features	*L. pneumophila*	*C. burnetii*	*Brucella spp*	*Bartonella spp*	Selected
1	AA composition	**1**	**2**	**3**	**1**	*
2	Auto-covariance of PSSM	**2**	**3**	**2**	**3**	*
3	PSSM composition	**3**	**1**	**1**	**2**	*
4	Dipeptide composition	**4**	**4**	**4**	**4**	*
5	Homology to known effectors	**1**	1	1	1	*
6	Average hydropathy	**2**	4	13	4	*
7	Total Hydropathy	**3**	6	8	5	*
8	Hydropathy of C terminal	**4**	3	21	23	*
9	Pepcoil hitcount	**5**	11	7	19	*
10	Hydropathy of N terminal	6	**5**	28	3	*
11	Pepcoil length	**7**	12	8	20	*
12	Charge of C terminal	8	35	3	9	
13	Coiled coil domain	**9**	**7**	11	22	*
14	Signal peptide probability	**10**	37	2	27	*
15	Polarity	**11**	29	29	15	*
16	Molecular mass	**12**	28	23	16	*
17	Maximum cleavage site probability	**13**	36	16	24	*
18	Transmembrane helices	14	14	30	14	
19	Length	**15**	15	24	18	*
20	Isoelectric point	**16**	30	25	17	*
21	Ank domain	17	10	31	28	
22	Basicity of N terminal	18	34	22	8	*
23	E-Block	19	18	10	28	
24	Coiled coils secondary structure	40	8	17	25	
25	*α* helices secondary structure	38	**2**	19	11	*
26	*β* strands secondary structure	24	9	26	2	
27	Transmembrane prediction by philius	35	13	20	6	
28	Total charge	21	39	18	7	
29	Charge of N terminal	23	17	27	10	
30	Basicity of C terminal	31	38	4	21	
31	Combined content of I, L, V and F	41	32	9	28	
32	Combined content of D and E	42	22	31	28	
33	Combined content of N and Q	28	33	31	28	
34	Combined content of R, K and H	37	25	6	28	
35	Combined content of S and T	43	27	31	28	
36	Combined content of S, N, E, and K	27	19	12	28	
37	Combined content of V, A, G and I	43	23	31	28	
38	protein subcellular localization	23	13	5	13	
39	DUF domain	33	26	15	28	
40	TM domain	25	16	14	12	
41	F-box domain	26	31	31	28	
42	F-box like domain	29	40	31	28	
43	U-box domain	34	40	31	28	
44	Pkinase domain	39	20	31	28	
45	LLR domain	43	40	31	28	
46	TPR domain	43	40	31	28	
47	Sel1 domain	32	21	31	28	
48	Patatin domain	22	40	31	28	
49	NLS domain	20	24	31	26	
50	MLS domain	36	40	31	28	
51	Prenylation domain	30	30	31	28	

The underlined features in the table represent features that were selected following our filtering feature selection method. As mentioned, filtering was performed using a p-value threshold determined by Bonferroni correction. Other features that are not selected but have the ranks less than 37, 31, 18, and 23 for *L. pneumophila*, *C. burnetii*, *Brucella spp*, and *Bartonella spp*, respectively, have p-values smaller than 0.5 and can be considered to have the potentiality of inclusion in prediction models.

Features given in blue were selected following the complete statistical approach shown in Fig 1 that concludes in the building of a logistic regression model. They are the set of features that have worked effectively as a group for predicting effector proteins. The selected features for *L. pneumophila*, which has the greatest number of known effectors, include almost all the selected features for the other three bacteria. Moreover, the elements of each vector feature, presented in S2 to S5 Tables, follow the same pattern. Based on the results presented, the final set of features, composed of the union of selected features (in blue) and marked by an asterisk in the last column of the table, are proposed for prediction of T4SS effectors. A complete list of these features is given in S6 Table.

As the different elements of the vector features were included in the set, we conclude that these vector features are important predictors for all of our pathogens. As we can see and as one might guess, homology to known effectors has a high rank as an effective feature for all four bacteria. In addition hydropathy-related features have high rankings in the table which shows that the degree of hydrophobicity of proteins plays an important role for effectors. The presence of coiled coil domains and protein length are also important indicators of effector proteins, and overall, the secondary structure of proteins seems to be important for effectors. Finally we can see that in addition to the chemical properties of a protein sequence, its structure and composition as well as its topology all have a share in determining whether a protein is an effector.

By considering the bottom part of Table 2, which shows features that were not selected for our four pathogens, we can conclude that some combinations of amino acids, with p-values in the range of 0.08 to 0.9 for *L. pneumophila*, as well as the presence of some domains, with p-values in the range of 0.005 to 0.9 for *L. pneumophila*, are not highly effective predictors of whether a protein is an effector. For example, some domains, such as Patatin and F-box, may be specific to certain bacteria or they may be present in a small subset of effector proteins. NLS (Nuclear Localization Signals), which target proteins to the nucleus of eukaryotic cells, were not selected as a highly effective feature, but NLS rank more highly for some of our bacteria compared to other features that were not selected. The same is true for MLS (Mitochondrial localization signals) which are signal sequences in the N-terminus of proteins that are targeted to the mitochondria. Also, total charge, charge of N-terminus, and basicity of C-terminus, with p-values in the range of 0.008 to 0.1 for *L. pneumophila*, were not as effective as other selected features.

As mentioned previously, hydropathy plays an important role in predicting effectors. In fact, from our table we see that all four hydropathy measures, with p-values on the order of 10^−33^ to 10^−12^ ($p\approx O\left(10^{-33}\right)-O\left(10^{-12}\right)$), which give both hydrophobic and hydrophilic characteristics of a protein sequence, are effective predictors of whether a protein is an effector, although hydropathy of the C-terminus appears to be more effective than hydropathy of the N-terminus. Hydropathy of effector proteins tends to be more negative than non-effectors. For example, for *L. pneumophila* the average hydropathy of effectors has a mean of approximately -40 compared to 0 for non-effectors. Also, total hydropathy, hydropathy of C-terminus, and hydropathy of N-terminus have averages of -194.5, -16.7, and -5.9, respectively, for effectors compared to -27.13, -6, and 2.9 for non-effectors.

The presence of coiled coil domains, structural motifs in a protein sequence ($p\approx O(10^{-13})$) for *L. pneumophila*, appears to have a significant impact on the probability of effector prediction as both features calculated using different methods have been selected in our feature set. For *L. pneumophila* the pepcoil hitcount (the number of coiled coil domains) and pepcoil length (the total length of coiled coil domains), the averages are 0.64 and 20.1 for effectors, respectively, compared to 0.1 and 3.9 for non-effectors. For *C. burnetii*, 9.9 and 3.2 are the average pepcoil lengths for its effectors and non-effectors, respectively. In addition, using Pfam and SMART tools shows that in *L. pneumophila* about 22% of effectors have coiled coil domains while only 4% of non-effectors contain one of these domains. In *C. burnetii*, it seems that secondary structure is more important for predicting effectors than for the other three bacteria as seen in Table 2.

Effector proteins appear to have a lower probability of having signal peptide cleavage sites. For *L. pneumophila* this feature has averages of 0.054 and 0.13 for effectors and non-effectors, respectively, ($p\approx O(10^{-7})$), and maximum cleavage site probability shows the same trend.

Next we consider some chemical properties of protein sequences. Effector proteins have higher polarity than non-effectors. For instance, for *L. pneumophila* the average polarity (using Grantham indices) for effectors and non-effectors is 3884 and 3005, respectively, ($p\approx O(10^{-7})$). Moreover, effectors are longer and have higher molecular mass than non-effectors. For example, for *L. pneumophila* the average lengths and molecular masses are 471 and 2831, respectively, for effectors and 377 and 2279 for non-effectors ($p\approx O(10^{-6})$). Ank domains function as protein-protein interaction domains. For *C. burnetii* about 14% of effector proteins contain an Ank domain while nearly no non-effectors do ($p\approx O(10^{-4})$). Thus, Ank domain presence appears to increase the probability of effector prediction. The same observation is made for E-Block, a domain which consists of a glutamate-rich sequence in the C-terminus of a protein. Our results indicate that about 6% of effectors in *L. pneumophila* have this domain, while almost none of the non-effectors do ($p\approx O(10^{-6})$).

Examination of our results and the predictions that we made in [10] for *C. burnetii*, indicate that we have identified a set of effective features for T4SS effector protein prediction.

## Conclusion

The final goal of this study was to find a set of optimal features for prediction of T4SS effectors. For this purpose, we worked with four types of pathogens and gathered validated sets of protein sequences of effectors and non-effectors for each type to create our datasets. Then by means of an extensive literature search we collected a set of features proposed in different works to be important in T4SS effector prediction. We calculated each feature for all protein sequences in our four datasets and evaluated their effectiveness using the *t*-test to filter less important ones. Then using PCA and factor analysis, we reduced the dimensions of the features set and eliminated correlation between features. Finally by creating logistic regression models, we selected a set of effective features that led to high accuracy for differentiating between effectors and non-effectors for each type of bacteria. Based on the set of selected features, we conclude that *L. pneumophila* features, which has the largest number of known effectors, include almost all the features selected for the other three pathogens.

Moreover, of all the features examined, the most important ones are vector features including the position specific scoring matrix (PSSM), amino acid composition, and dipeptide composition. In addition, some chemical properties as well as topology related features such as hydropathy and coiled coil domains are also important.

In future work, the final set of selected features can be used to develop a machine learning algorithm for prediction of T4SS effectors for different types of pathogens.

## Supporting information

S1 Table Complete list of features used in this work. (XLSX)

S2 Table Selected features after logistic regression modeling for *L. pneumophila*. (XLSX)

S3 Table Selected features after logistic regression modeling for *C. Burnetii*. (XLSX)

S4 Table Selected features after logistic regression modeling for *Brucella* spp. (XLSX)

S5 Table Selected features after logistic regression modeling for *Bartonella* spp. (XLSX)

S6 Table Set of features selected for prediction of T4SS effectors. (XLSX)

S1 File Set of known effectors for *L. pneumophila*. (FASTA)

S2 File Set of known effectors for *C. Burnetii*. (FASTA)

S3 File Set of known effectors for *Brucella* spp. (FASTA)

S4 File Set of known effectors for *Bartonella* spp. (FASTA)

S5 File Set of non-effectors for *L. pneumophila*. (FASTA)

S6 File Set of non-effectors for *C. Burnetii*. (FASTA)

S7 File Set of non-effectors for *Brucella* spp. (FASTA)

S8 File Set of non-effectors for *Bartonella* spp. (FASTA)
